# DHA- and EPA-Enriched Phosphatidylcholine Suppress Human Lung Carcinoma 95D Cells Metastasis via Activating the Peroxisome Proliferator-Activated Receptor γ

**DOI:** 10.3390/nu14214675

**Published:** 2022-11-04

**Authors:** Haowen Yin, Yuanyuan Liu, Hao Yue, Yingying Tian, Ping Dong, Changhu Xue, Yun-Tao Zhao, Zifang Zhao, Jingfeng Wang

**Affiliations:** 1 College of Food Science and Engineering, Ocean University of China, Qingdao 266003, China; 2 Marine Biomedical Research Institute of Qingdao, Qingdao 266071, China; 3 Laboratory for Marine Drugs and Bioproducts, Pilot National Laboratory for Marine Science and Technology, Qingdao 266237, China; 4 College of Food Science and Technology, Guangdong Ocean University, Zhanjiang 524088, China; 5 Hainan Huayan Collagen Technology Co., Ltd., Haikou 571000, China

**Keywords:** DHA-PC, EPA-PC, human lung carcinoma 95D cells, PPARγ

## Abstract

The antineoplastic effects of docosahexaenoic acid-containing phosphatidylcholine (DHA-PC) and eicosapentaenoic acid-containing phosphatidylcholine (EPA-PC) were explored, and their underlying mechanisms in the human lung carcinoma 95D cells (95D cells) were investigated. After treatment of 95D cells with DHA-PC or EPA-PC, cell biological behaviors such as growth, adhesion, migration, and invasion were studied. Immunofluorescence and western blotting were carried out to assess underlying molecular mechanisms. Results showed that 95D cells proliferation and adherence in the DHA-PC or EPA-PC group were drastically inhibited than the control group. DHA-PC and EPA-PC suppressed the migration and invasion of 95D cells by disrupting intracellular F-actin, which drives cell movement. The protein expression of PPARγ was induced versus the control group. Furthermore, critical factors related to invasion, including matrix metallopeptidase 9 (MMP9), heparanase (Hpa), and vascular endothelial growth factor (VEGF), were drastically downregulated through the PPARγ/NF-κB signaling pathway. C-X-C chemokine receptor type 4 (CXCR4) and cofilin were significantly suppressed via DHA-PC and EPA-PC through the PPARγ/phosphatase and tensin homolog (PTEN)/serine-threonine protein kinase (AKT) signaling pathway. DHA-PC and EPA-PC reversed the PPARγ antagonist GW9662-induced reduction of 95D cells in migration and invasion capacity, suggesting that PPARγ was directly involved in the anti-metastasis efficacy of DHA-PC and EPA-PC. In conclusion, DHA-PC and EPA-PC have great potential for cancer therapy, and the antineoplastic effects involve the activation of PPARγ. EPA-PC showed more pronounced antineoplastic effects than DHA-PC, possibly due to the more robust activation of PPARγ by EPA-PC.

## 1. Introduction

Cancer incidence and mortality are rapidly growing worldwide, along with increases in socioeconomic development. The International Agency for Research on Cancer announced the addition of 19.2 million cancer cases worldwide in 2020, which has become a significant barrier to longevity [1,2]. The vast majority of cancer-related deaths are attributed to metastatic disease rather than to primary tumor growth [3]. Once cancer becomes advanced, metastatic cancer cells might quickly develop in other organs and/or tissues, making cancer more difficult to treat [4,5]. Cancer metastasis involves a complex series of interrelated processes. The tumor cells separate from the primary tumor, adhere to endothelial cells, and degrade the extracellular matrix (ECM) [6,7]. Invasive tumor cells escape the immune system, infiltrate the vascular network, travel within the vascular network, and then form new tumors [8,9]. Antitumor metastasis research has received enormous attention and has become an essential field in antitumor drug research.

According to epidemiological studies, the type and quantity of fatty acids consumed by humans were negatively related to the risk of tumor onset [10]. The main source of n-3 polyunsaturated fatty acids is seafood, and its prominent members are eicosapentaenoic acid (EPA) and docosahexaenoic acid (DHA). The paper reported that cancer patients increased their intake of EPA and DHA to reduce the incidence of remote tumor metastasis [11,12]. Several papers have exhibited that DHA and EPA could affect cancer cell proliferation, apoptosis, angiogenesis, migration, and invasion [13,14]. Therefore, the effects of DHA and EPA on the prevention and treatment of cancer are of interest. However, DHA and EPA are highly sensitive to free radicals and reactive oxygen and easily oxidized to toxic oxidation products such as hydroperoxides and aldehydes [15]. N-3 PUFAs in the form of phospholipids, as a new generation of omega-3 lipids, have recently gained attention for their better bioavailability and antioxidant properties [16,17]. However, the effects of n-3 PUFAs in the form of phospholipids on tumor development and progression have rarely been reported.

PPARγ is a part of the nuclear hormone receptor superfamily [18]. PPARγ combines with the retinoic acid receptor (RXR) to form a functional heterodimer, and then the heterodimer enters the nucleus, where it associates with a peroxisome proliferator response element (PPRE) within a target gene to initiate transcription [19]. Recent research has shown that activation of PPARγ can inhibit cancer cell proliferation, promote cell cycle arrest, reduce cancer cell migration and invasion, and inhibit angiogenesis [20,21,22,23]. These studies indicate that PPARγ might be a relevant target for cancer therapy.

In this study, the two characteristic phospholipids containing n-3 PUFAs were prepared, DHA-containing phosphatidylcholine (DHA-PC) and EPA-containing phosphatidylcholine (EPA-PC). Here, the effects of DHA-PC and EPA-PC on metastasis in the highly metastatic 95D cells line were investigated. Meanwhile, mechanisms underlying the PPARγ in the anti-metastasis efficacy of DHA-PC and EPA-PC were evaluated.

## 2. Materials and Methods

### 2.1. Reagents

Squid roes (*S. oualaniensis*) and sea cucumbers (*Cucumaria frondosa*) were provided by Tuandao (Qingdao, China). RPMI 1640 medium was provided by Gibco (New York, NY, USA). FBS was bought from Zeta Life (San Francisco, CA, USA). The lactate dehydrogenase (LDH) kit was donated by Jiancheng (Nanjing, China). GW9662 was donated by Sigma-Aldrich (St. Louis, MO, USA). The anti-PPARγ, anti-retinoid X receptor alpha (RXRα), anti-phosphatase and tensin homolog (PTEN), anti-phosphoinositide 3-kinase (PI3K), anti-serine-threonine protein kinase (AKT), anti-phosphorylated AKT(p-AKT), anti-C-X-C chemokine receptor type 4 (CXCR4), and anti-cofilin antibodies were provided by Abcam (Burlingame, CA, USA). The anti-Matrix metallopeptidase 9 (MMP9), anti-metallopeptidase inhibitor 1 (TIMP1), anti-TGF-β, and anti-cofilin antibodies were obtained from CST (Danvers, MA, USA). The anti-NF-κB and anti-phosphorylated NF-κB (p-NF-κB) antibodies were bought from Santa Cruz (Dallas, TX, USA). The anti-vascular endothelial growth factor (VEGF), anti-heparanase (Hpa), and anti-F-actin antibodies were acquired from Abcam (Cambridge, UK).

### 2.2. Preparation of DHA-PC and EPA-PC

DHA-PC was extracted from squid roes, and EPA-PC was extracted from the sea cucumbers, according to previous papers by our laboratory [24]. Briefly, the homogenized raw material was thoroughly mixed with chloroform and NaCl solution (0.15 mol/L) and allowed to stand for 24 h to remove chloroform to obtain total lipids. The PC was eluted from neutral lipids and glycolipids using silica gel column chromatography with acetone, chloroform, and methanol in sequence. The eluate was collected and dried using a rotary evaporator.

### 2.3. Preparation of the Liposome

Cholesterol and equimolar amounts of DHA-PC and EPA-PC were dissolved in chloroform and spin-distilled according to literature methods [25]. The liposomes are mixed with distilled water and charged with nitrogen. Lipid films were obtained by evaporation under reduced pressure. The liposome suspension was sonicated and homogenized for 1 h at 4 °C, and homogeneous liposomes were extruded through a liposome extruder.

### 2.4. Cell Culture and Treatment

Human lung carcinoma 95D cells (95D cells) and human umbilical vein endothelial cells (HUVECs) were donated via the Procell (Wuhan, China). The method of 95D cells and HUVECs culture and treatment was suitably modified based on the previous protocols [26,27]. 95D cells and HUVECs were respectively cultured in the RPMI 1640 medium and F-12K medium (10% FBS and 1% penicillin/streptomycin solution). Meanwhile, cells were supported in a humid environment at 37 °C with 5% CO_2_.

### 2.5. Cell Viability and Cytotoxicity Assay

The 95D cells in the logarithmic growth phase were adjusted to 10^4^ cells/mL by the complete medium. 95D cells were inoculated in cell culture plates (100 μL/well) and cultured for 24 h. A complete medium containing different concentrations of DHA-PC or EHA-PC (0, 12.5, 25, 50, 100, and 200 μg/mL) was replaced and cultured for 24 h and 48 h. Cell viability assay was measured via the MTT method, and OD_540_ values were obtained. The supernatant of the cell culture medium was collected from 48 h of culture, and the LDH content in the supernatant was measured by an LDH assay kit. The operating procedures followed the instruction manual.

### 2.6. Monolayer Cell Adhesion Assay

For the ECM adhesion assay, 95D cells were inoculated in 24-well plates at a concentration of 3 × 10^4^ cells/mL and plastered for 24 h. The medium was discarded, and a complete medium containing different concentrations of DHA-PC or EPA-PC (50 μg/mL and 100 μg/mL) was added for 24 h. The cells were added at 100 μL/well to pre-coated matrigel plates and incubated for 1 h. The unadhered cells were washed off by adding D-HBSS, and the OD_570_ values were determined by the MTT method. HU-VECs were inoculated in cell culture plates for the endothelial cell adhesion assay and maintained in a complete medium until 95% confluence was reached. 95D cells (5 × 10^4^/well) were added to the monolayer HUVECs. After 30 min incubation, cell suspensions were removed, the unadhered cells were washed off by adding D-HBSS, and the OD_570_ values were determined by the MTT method. The remaining cells were randomly counted in at least 5 fields under the light microscope, and the images were collected via an inverted microscope. The experimental protocol referred to in the previous studies with slightly adjusted accordingly [27,28].

### 2.7. Migration Assays and Invasion Assays

The migration of 95D cells was determined via 24-well plates with Transwell inserts. 95D cells were incubated with the extracted compound for 24 h and adjusted to 2.5 × 10^5^ cells/mL by the serum-free medium. 200 μL of the well-mixed cell suspension was aspirated into the upper chamber. 600 μL of complete medium was aspirated into the lower chamber of the Transwell, incubated in the incubator for 24 h, and then strained using crystal violet. The upper chamber of the 24-well Transwell was pre-coated with Matrigel, and then 200 μL of the well-mixed cell suspension was aspirated into the upper chamber, while 600 μL of complete medium was aspirated into the lower chamber and incubated in the incubator for 24 h. Non-invasive cells were carefully removed from the upper chamber, and the 95D cells in the lower chamber were stained with crystal violet. Images from at least 5 fields of each well were randomly selected and evaluated via Image Pro Plus 6.0 software. The experimental protocol was based on the previous studies with appropriately modified [29,30].

### 2.8. Immunofluorescence

The 95D cells (10^5^ cells/well) were randomly seeded in 6-well cell culture plates containing coverslips and incubated for 12 h. Then 95D cells were kept in media supplemented with DHA-PC or EPA-PC (100 μg/mL) for 36 h. After D-HBSS washing, the 95D cells were fixed with 4% paraformaldehyde for 40 min. The coverslips were removed from the plates and reacted with the anti-F-actin (1:100) antibody in a wet box for 1 h. The coverslips were washed in PBST for 6 min to remove unbound antibodies. Additionally, the coverslips were reacted with Alexa-labeled IgG secondary antibody (1:1000) for 1 h. DAPI was used for nuclei staining for 1 min. Images were photographed under a fluorescence confocal microscope. The experimental method referred to the previous studies with appropriately modified [31,32].

### 2.9. Western Blotting Analysis

According to the previous protocols with appropriately modified [33]. Briefly, the total protein was sequentially separated via 12% SDS-PAGE and PVDF membrane. The PVDF membrane was incubated with antibodies for 6 h in a low-temperature environment. The membrane was subsequently maintained with horseradish peroxidase-conjugated secondary antibody for 1.5 h. After D-HBSS washing, Tanon 5200 chemiluminescence instrument (Shanghai, China) was used to visualize the images. Western blotting was normalized relative to the β-actin [34,35].

### 2.10. Statistical Analysis

All data were exhibited as mean ± SD of three independent experiments. Statistical comparisons were tested using ANOVA analysis followed by Bonferroni’s multriple range *post-hoc* analysis using SPSS 21.0 software (IBM, Armonk, NY, USA). *p*-value <0.05 was considered obviously significant.

## 3. Results

### 3.1. Effect of DHA-PC and EPA-PC on 95D Cell Proliferation

**Figure 1:**
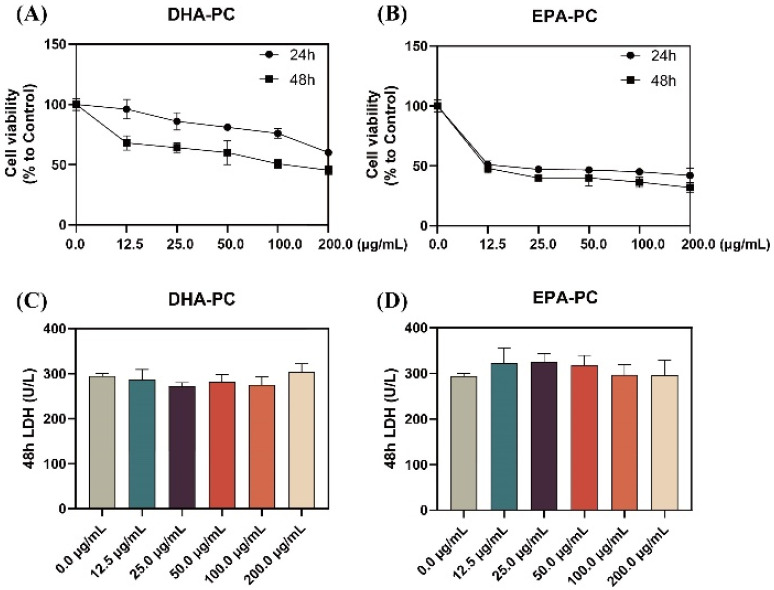
DHA-PC and EPA-PC inhibited 95D cells proliferation and exhibited non-toxicity to 95D cells. Evaluation of the effect of various concentrations of DHA-PC (**A**) and EPA-PC (**B**) on the cell viability of 95D cells. Evaluation of the effect of different concentrations of DHA-PC (**C**) and EPA-PC (**D**) on the LDH activity of 95D cells.

As shown in Figure 1A,B, the proliferation of 95D cells was significantly limited with increasing doses of DHA-PC or EPA-PC during the 24 h and 48 h interventions of the extracted compound, with EPA-PC showing better inhibition (IC_50_, 9.9 μg/mL). Extracellular LDH release is a marker of cell cytotoxicity. The LDH content was measured in the supernatant of 95D cells after receiving the extracted compound for 48 h. The results showed that the extracted compound did not notably exhibit toxicity to 95D cells (Figure 1C,D). Based on the proliferation inhibition results, the groups (50 μg/mL and 100 μg/mL) were selected for subsequent experiments.

### 3.2. Effect of DHA-PC and EPA-PC on the Adhesion of 95D Cells to ECM and HUVECs

**Figure 2:**
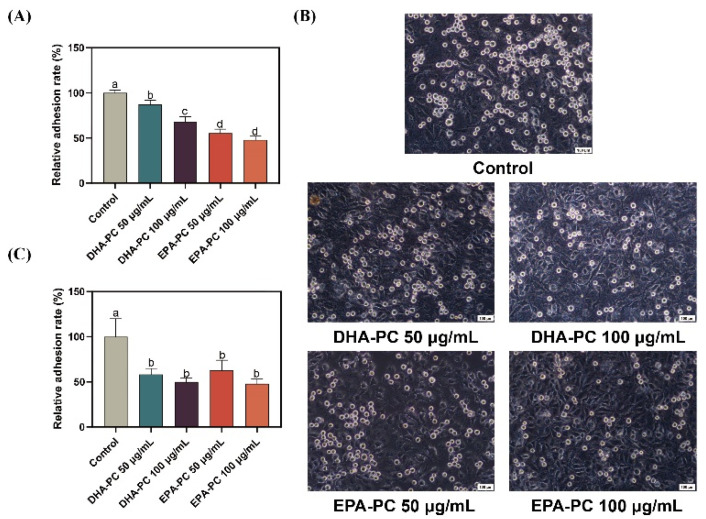
DHA-PC and EPA-PC suppressed the adhesion of 95D cells to ECM and HUVECs. (**A**) Quantification of 95D cells adhered to the Matrigel. (**B**) The representative images of 95D cells adhered to the HUVECs monolayers (Scale bar: 100 μm). (**C**) Quantification of 95D cells adhered to the HUVECs monolayers. Different letters (a, b, c, and d) indicate remarkable differences at *p* < 0.05 among all groups determined by ANOVA (Bonferroni’s test).

Cancer cell adhesion underlying cell-ECM and cell-cell interactions contribute to tumor cell metastasis [36]. Firstly, the adhesion of 95D cells treated with DHA-PC or EPA-PC to the ECM was evaluated (Figure 2A). Compared to the control group, 95D cells in the DHA-PC and EPA-PC groups were suppressed ECM adhesion. Moreover, the adhesion ability of 95D cells to HUVECs was examined. As Figure 2B,C show, 95D cells in the DHA-PC and EPA-PC groups showed markedly less adherence to the HUVECs than the control cells, respectively. These data exhibited that DHA-PC and EPA-PC suppress the adhesion ability of 95D cells, and EPA-PC exerts a more substantial effect than DHA-PC.

### 3.3. Effect of DHA-PC and EPA-PC on the Migration and Invasion Ability of 95D Cells

**Figure 3:**
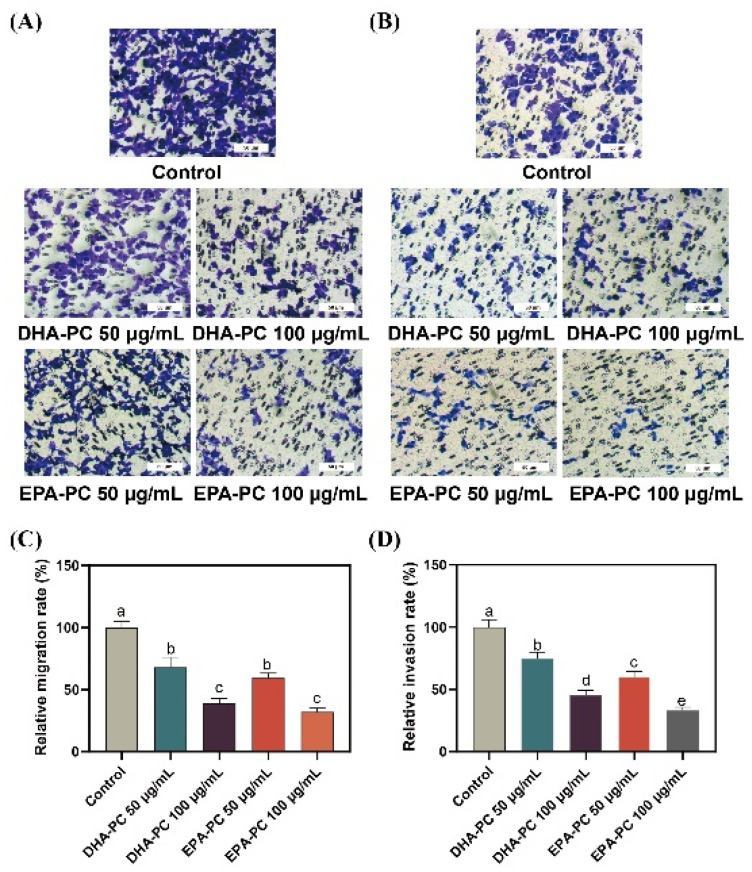
DHA-PC and EPA-PC suppressed the migration and invasion ability of 95D cells. (**A**) The migrated cells were photographed under the microscope (Scale bar: 50 μm). (**B**) The invaded cells photographed were under the microscope (Scale bar: 50 μm). (**C**) Quantitative analysis of migrating cells. (**D**) Quantitative analysis of invaded cells. Different letters (a, b, c, d, and e) indicate remarkable differences at *p* < 0.05 among all groups determined by ANOVA (Bonferroni’s test).

Compared to 95D cells in the control group, cells treated with the DHA-PC or EPA-PC were showed significant inhibitory effects on migrating cells, respectively (Figure 3A,C). Similarly, the extracted compound reduced the invasion of 95D cells in a concentration-dependent manner, respectively (Figure 3B,D). The results suggest that the extracted compound possess potential anti-metastasis activity by suppressing 95D cells migration and invasion. For the study of the possible mechanism of DHA-PC and EPA-PC on 95D cells metastasis, a high-dose group (100 μg/mL) was chosen for subsequent research.

### 3.4. Effect of DHA-PC and EPA-PC on F-Actin Organization through the TGF-β Pathway

**Figure 4:**
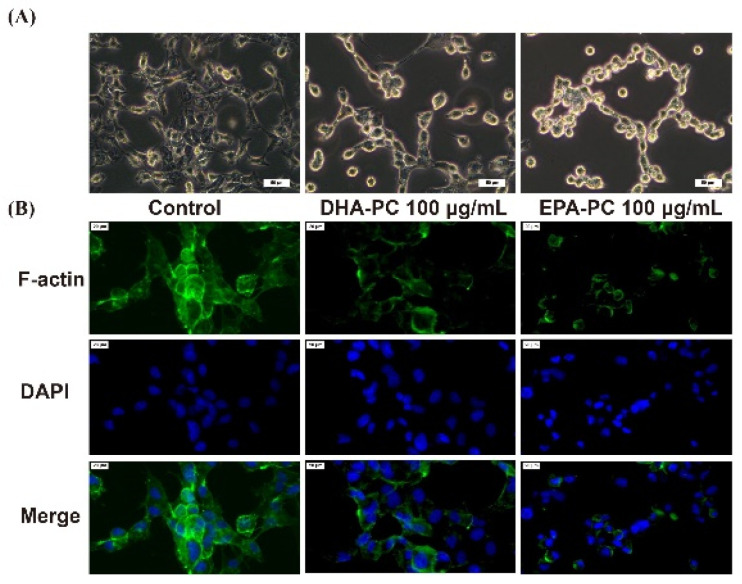
DHA-PC and EPA-PC destroyed the F-actin organization of 95D cells. (**A**) The morphological change of 95D cells was taken under the optical microscope (Scale bar: 50 μm). (**B**) Immunofluorescence analysis was presented using the fluorescence microscope (Scale bar: 20 μm).

The invasive and migration abilities of cancer cells are highly related to the cell cytoskeleton [26]. Therefore, the distribution of intracellular F-actin in 95D cells treated with DHA-PC or EPA-PC was investigated. After intervention with the DHA-PC or EPA-PC, intracellular F-actin staining became unclear, degraded, and fragmented versus the control group (Figure 4). The data presented that 100 μg/mL of the extracted compound could control cell motility via destroying intracellular F-actin and that EPA-PC exerts a better substantial disruptive effect vs. DHA-PC.

### 3.5. Effect of DHA-PC and EPA-PC on PPARγ

**Figure 5:**
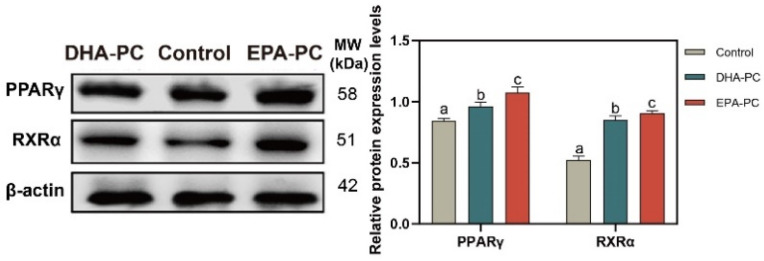
DHA-PC and EPA-PC increased the protein levels of PPARγ and RXRα. The expression of PPARγ and RXRα. Different letters (a, b, and c) indicate remarkable differences at *p* < 0.05 among all groups determined by ANOVA (Bonferroni’s test).

The protein expression of PPARγ and RXRα were tested to determine whether DHA-PC or EPA-PC exhibited antitumor effects through the activation of PPARγ. The protein expression of PPARγ and RXRα were remarkably enhanced versus the control group (Figure 5), respectively. EPA-PC expressed a more substantial effect than DHA-PC.

### 3.6. Effect of DHA-PC and EPA-PC on the Expression of Crucial Factors Related to Invasion through the NF-κB Pathway

**Figure 6:**
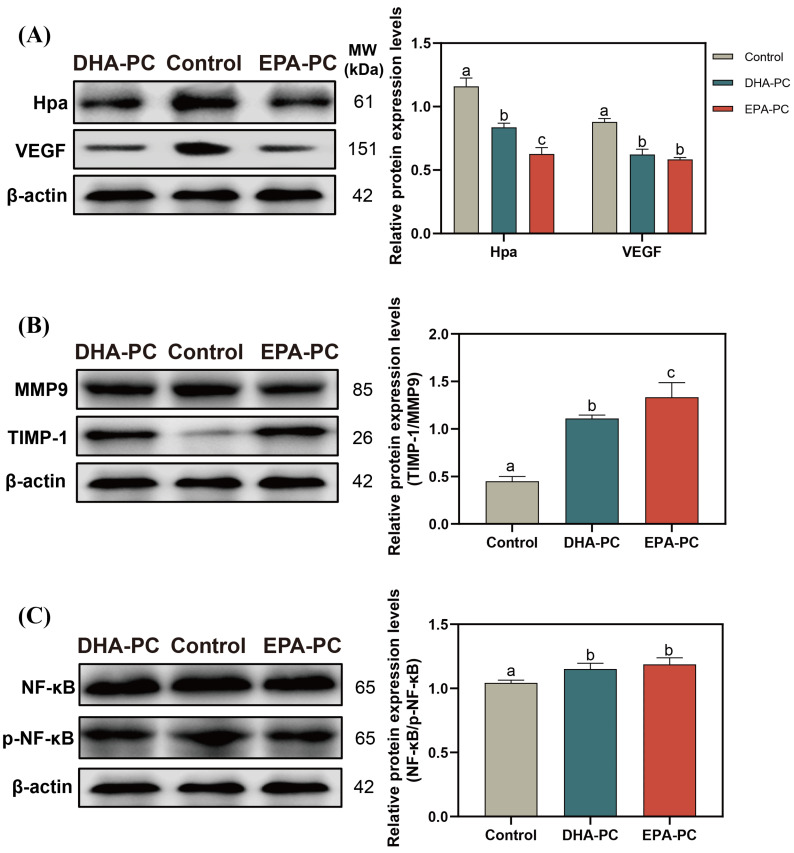
DHA-PC and EPA-PC suppressed protein expression of crucial transcription factors of the invasion and adhesion. (**A**) the expression of Hpa and VEGF. (**B**) the expression of MMP9 and TIMP-1. (**C**) the expression of NF-κB and p-NF-κB. Different letters (a, b, and c) indicate remarkable differences at *p* < 0.05 among all groups determined by ANOVA (Bonferroni’s test).

Hpa destroys the integrity of ECM and promotes the release of VEGF [37], which is conducive to the invasion of the cancer cell. After the extracted compound intervention, the protein expression of Hpa and VEGF was remarkably decreased (Figure 6A), and the TIMP1/MMP9 level was significantly upregulated compared to the control group (Figure 6B), respectively. 100 μg/mL of DHA-PC or EPA-PC considerably suppressed the p-NF-κB expression in the 95D cells versus the control group (Figure 6C), respectively. These above results showed that the extracted compound could suppress the invasion of 95D cells via the PPARγ/NF-κB pathway.

### 3.7. Effect of DHA-PC and EPA-PC on the Expression of Key Factors Related to Proliferation and Migration through the PTEN/AKT Pathway

**Figure 7:**
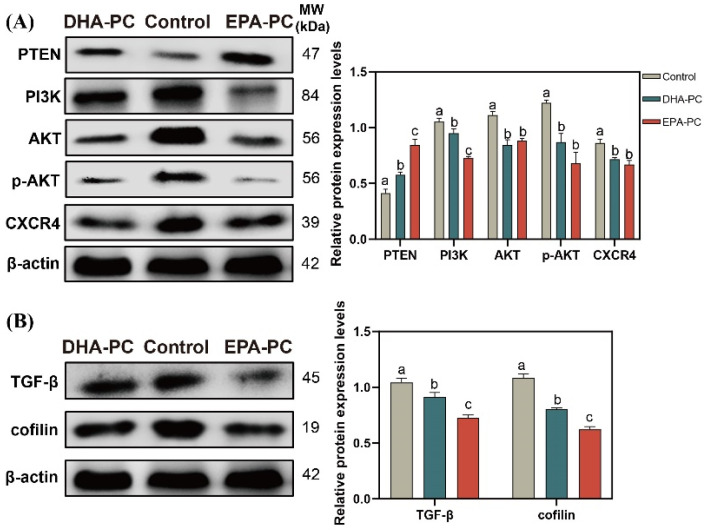
DHA-PC and EPA-PC downregulated the protein level of key transcription factors of the migration. (**A**) the expression of PTEN, PI3K, AKT, p-AKT, and CXCR4. (**B**) the expression of TGF-β and cofilin. Different letters (a, b, and c) indicate remarkable differences at *p* < 0.05 among all groups determined by ANOVA (Bonferroni’s test).

The tumor suppressor PTEN can be positively regulated by PPARγ [22]. The expression of PTEN was considerably increased after the extracted compound intervention, respectively (Figure 7A). EPA-PC exhibited an enormous increase in PTEN expression, consistent with the finding that EPA-PC showed greater activation of PPARγ. The analysis exhibited that the protein expression of PI3K, AKT, p-AKT, and CXCR4 were obviously downregulated (Figure 7A) versus the control group. Cofilin is involved in the F-actin polymerization and reorganization [38]. As shown in Figure 7B, the data exhibited that the protein expression of cofilin and TGF-β in the extracted compound group was obviously reduced versus the control group.

### 3.8. Effect of DHA-PC and EPA-PC on 95D Cell Migration and Invasion via PPARγ Activation

**Figure 8:**
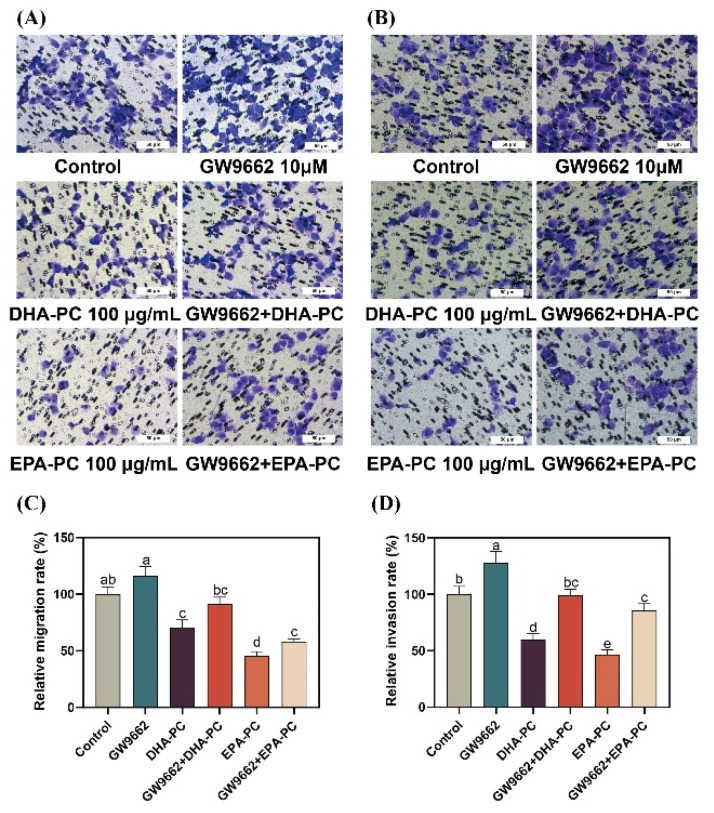
PPARγ-specific antagonist reversed the effects of regarding the suppression of 95D cell migration and invasion by DHA-PC and EPA-PC. (**A**) The migrated cells were photographed under the microscope (Scale bar: 50 μm). (**B**) The invaded cells were photographed under the microscope (Scale bar: 50 μm). (**C**) Quantitative analysis of migrating cells. (**D**) Quantitative analysis of invaded cells. Different letters (a, b, c, d, and e) indicate remarkable differences at *p* < 0.05 among all groups determined by ANOVA (Bonferroni’s test).

To further explore the role of PPARγ activation in DHA-PC- and EPA-PC-induced anti-metastasis effects, 95D cells were incubated with a PPARγ-specific antagonist (GW9662). 95D cells in the GW9662 group was remarkably increased migratory ability (Figure 8A) than the control group. The extracted compound administration rescued these changes drastically. Similarly, the extracted compound intervention reversed the invasion inhibition of 95d cells caused by GW9662 (Figure 8B). The data exhibited that the extracted compound led to the inhibition of migration and invasion by activating PPARγ.

## 4. Discussion

Globally, cancer imposes a considerable burden on families and affects physical and mental health. Antitumor metastasis research has received enormous attention and has become an essential field in antitumor drug research. In this study, DHA-PC and EPA-PC effect on 95D cells metastasis was investigated. The results found that DHA-PC and EPA-PC inhibited the proliferation, adhesion, migration, and invasion of 95D cells. Meanwhile, DHA-PC and EPA-PC destroyed the F-actin organization of cancer cells. Further mechanistic studies revealed that DHA-PC and EPA-PC could activate PPARγ to suppress the metastasis and proliferation of 95D cells.

Extensive research has shown that DHA and EPA exhibit anti-cancer efficacy through anti-inflammation mechanisms [13,39]. DHA and EPA could be transformed into leukotriene and prostaglandin after being absorbed and affect cancer activity through the cyclooxygenase pathway [40]. However, EPA-PC and DHA-PC are amphiphilic phospholipids that can enter cells directly through the cell membrane. The effects of DHA-PC and EPA-PC on cancer might not be limited to their anti-inflammatory roles. The invasive and migration abilities of cancer cells are highly related to the cell cytoskeleton, which consists of F-actin and microtubules [26,41]. The morphology of 95D cells failed to present stereoscopic outlines after treatment with DHA-PC or EPA-PC, which is consistent with previous papers that F-actin polymerization could directly drive cell movement [38]. Cofilin is essential for modulating actin polymerization. TGF-β signaling has been presented to induce EMT in the cancer cell, a transition closely related to cancer metastasis. The non-Smad signaling of TGF-β induces changes in actin dynamics by activating RhoA and the downstream signaling effectors LIM domain kinase 2 and cofilin [42,43]. DHA-PC and EPA-PC promoted the disorganization of F-actin by attenuating the TGF-β pathway. It is reported that PPARγ plays an integral part in the development and treatment of tumors [44]. Previous papers have reported that DHA and EPA exhibit antitumor effects that could be explained through the activation of PPARγ [21,39]. In accordance with this idea, our results suggest that DHA-PC and EPA-PC upregulate the PPARγ expression. Especially in the presence of a PPARγ inhibitor GW9662, the anti-metastasis efficacy of DHA-PC and EPA-PC on the 95D cells are partly abolished, indicating that DHA-PC and EPA-PC suppress metastatic properties partially through activation of PPARγ.

The essential step in the process of cancer cell invasion is the degradation of ECM components [6,45]. The literature has reported that elevated HPA activity (an endogenous β-glucuronidase) leads to an altered tissue microenvironment, reduced intercellular barrier function, and facilitated tumor infiltration and metastasis [37]. MMP9 not only contributes to the degradation of ECM but also to the release of hepatocyte growth factor, VEGF, and enhances tumor cell tolerance to stimulate tumor cell metastasis and angiogenesis [24,46]. As the primary natural inhibitor of MMP9, TIMP1 inhibits MMP9 activity by binding to MMP9 at a 1:1 ratio to form a complex [47]. This study found that Hpa was expressed at drastically lower levels after the extracted compound treatment. Furthermore, lower MMP9 expression and higher TIMP1 expression were induced by DHA-PC and EPA-PC.

In cancer cells, the protein overexpression of NF-κB has been associated with the promotion of invasion and migration by increasing its downstream NF-κB targets, such as MMP9, Hpa, and VEGF [37,48]. Previous studies have reported that PPARγ resists NF-κB-mediated gene expression by reducing NF-κB binding with p65 and p50 [39,49]. The data exhibited that the extracted compound attenuates the crucial invasion factors expression through the downregulation of NF-κB transcriptional activity. Additionally, the high expression of the PI3K/AKT pathway in tumor patients has been studied, and this overexpression accelerates the migration and survival of cancer cells, resulting in the formation of metastasis nodules [50]. Anti-oncogene PTEN induces apoptosis, suppression of cell growth, and arrestation of the cell cycle via reducing the PI3K/AKT pathway [51]. Further studies also reported that PPARγ activation increases functional PTEN protein levels [52]. Similarly, we observed that the extracted compound upregulated PTEN expression with the activation of PPARγ. At the same time, higher PTEN expression was negatively concerned with the phosphorylation of AKT. Our results indicate that PPARγ acts as a tumor suppressor by upregulating PTEN transcription. Studies have shown that the CXCL12-CXCR4 axis plays an essential part in the progression of more than 20 different cancers [53]. The protein highly expressed of CXCR4 in 95D cells results in accelerated migratory and invasive ability [54], and 95D cells with CXCR4 knockdown formed fewer lung metastases compared with CXCR4-positive cells [55]. Here, we found that the expression of CXCR4 was downregulated and accompanied by a decrease of p-AKT after intervention with the extracted compound, similar to previous research that showed overexpression of PTEN dramatically attenuates tumor expansion in the lung via AKT/CXCR4 signaling [56].

## 5. Conclusions

**Figure 9:**
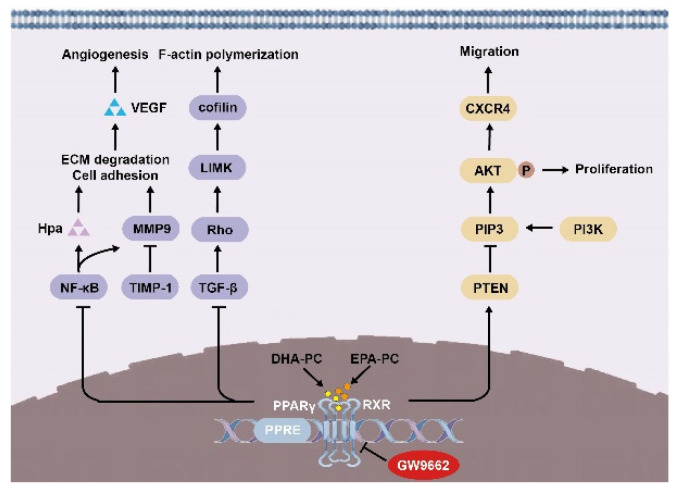
Schematic representation of the mechanisms by DHA-PC and EPA-PC suppresses 95D cells metastasis.

In summary, we demonstrated in 95D cells that DHA-PC and EPA-PC effectively inhibited proliferation, adherence, migration, invasive capability, and disrupted intracellular F-actin. Mechanically, DHA-PC and EPA-PC activated PPARγ, and PPARγ directly regulated the activity of tumor-related factors to achieve the antitumor effects (Figure 9). These findings provide a new perspective on the effects of DHA-PC and EPA-PC on cancer treatment, specifically on their role in the activation of PPARγ. Alternatively, DHA-PC and EPA-PC might be used as practical components applied to exceptional medical food for cancer patients.

## Data Availability

The original contributions presented in the study are included in the article, further inquiries can be directed to the corresponding authors.
